# Phylogenetic analysis of newly isolated grass carp reovirus

**DOI:** 10.1186/2193-1801-3-190

**Published:** 2014-04-15

**Authors:** Xiu-ying Yan, Ya Wang, Ling-fang Xiong, Ji-chang Jian, Zao-he Wu

**Affiliations:** Guangdong Key Laboratory of Pathogenic Biology and Epidemiology for Aquatic Economic Animals, Guangdong Ocean University, Huguangyan East, Zhanjiang, 524088 China; Zhongkai University of Agriculture and Engineering, Guangzhou, 510225 China

**Keywords:** Grass carp reovirus (GCRV), Identification, Phylogenetic relationship, Genotype

## Abstract

Grass carp reovirus (GCRV) is a causative agent of haemorrhagic disease in grass carp that drastically affects grass carp aquaculture. Here we report a novel GCRV isolate isolated from sick grass carp that induces obvious cytopathic effect in CIK cells and name it as GCRV096. A large number of GCRV 096 viral particles were found in the infected CIK cells by electron microscope. The shape, size and the arrangement of this virus were similar to those of grass carp reovirus. With the primers designed according to GCRV 873 genome sequences, specific bands were amplified from sick grass carp and the infected CIK cells. The homology rates among *vp4*, *vp6* and *vp7* gene in GCRV 096 and those of some GCRV isolates were over 89%. In this study, the sequences of *vp4*, *vp6* and *vp7* were used to analyse sequence variation, phylogenetic relationships and genotypes in twenty five GCRV isolates. The results indicated these twenty five GCRV isolates should be attributed to four genotypes. And there were no obvious characteristics in the geographical distribution of GCRV genotype. The study should provide the exact foundation for developing more effective prevention strategies of grass carp haemorrhagic disease.

## Introduction

Grass carp reovirus (GCRV), which is known as a member of the *Aquareovirus* genus and the Reoviridae family, can cause serious haemorrhagic disease in grass carp (Chen and Jiang 1983) and obvious cytopathic effect (CPE) on many cell lines from fish (Zuo et al. 1986; Lu et al. 1990). To date, a number of various GCRV isolates have been isolated from diseased grass carp around the world, including GCRV 873, GCRV 875, GCRV HZ08, GCRV GD108, AGCRV and others (Fang et al. 2002; Chi et al. 2011; Ye et al. 2012; Zeng et al. 2013). These isolates are distinct not only in their levels of virulence and cell culture characteristics, but also in their antigenicity (Fang et al. 2002; Mohd Jaafar et al. 2008; Zhang et al. 2010a).

GCRV is a double-stranded RNA virus that is assigned to the *Aquareovirus C* species. The genome of GCRV is known to consist of 11 segments of dsRNA contained in a core surrounded with a double-layered icosahedral capsid (Rangel et al. 1999). To our knowledge, there are few published reports about the serotype and genotype of GCRV. Furthermore, there are no uniform criteria for virus genotyping. One of the virus genotyping methods is based on the analysis of the nucleotide sequence.

So far, some gene sequences of GCRV isolates have been reported (Mohd Jaafar et al. 2008; Rangel et al. 1999; Fang et al. 2000; Su et al. 2010; Attoui et al. 2002; Fan et al. 2010). *vp4*, *vp6* and *vp7* gene in GCRV encode major outer capsid proteins and are conservative. Moreover, there are many variable sites and informative sites between sequences of *vp4*, *vp6* and *vp7* gene in different GCRV isolates. Considering *vp4*, *vp6* and *vp7* gene as molecular makers, we investigated sequence variation characteristics, the phylogenetic relationships and genotypes of twenty five GCRV isolates to find the evolutive characteristic of GCRV in the study. In this study, a new GCRV isolate was found and identified from diseased grass carp. This study provides the theoretical basis for the prevention and treatment of haemorrhagic disease in grass carp.

## Materials and methods

### Virus and cells

GCRV 096 was isolated from the diseased grass carp in Xiaogan, Hubei Province and stored in our laboratory. A widely used GCRV sensitive cell steain, grass carp kidney cells (CIK) were purchased from shenzhen inspection and quarantine bureau in China. CIK is GCRV sensitive cell and are widely used in the related study of GCRV (Ye et al. 2012; Zhang et al. 2010b; Ma et al. 2011).

Some GCRV isolates were examined in the present study, which were identified in previous studies (Mohd Jaafar et al. 2008; Rangel et al. 1999; Fang et al. 2000; Su et al. 2010; Attoui et al. 2002; Fan et al. 2010). Table 1 presents information about the specific names of twenty five GCRV isolates, their abbreviations, locations where they were collected, the genes of GCRV and their GenBank accession numbers.

**Table 1:** **Names of GCRV isolates, abbreviations, localities, genes of GCRV used in this study and their GenBank accession numbers**

Names	Abbreviations	Localities	Genes	GenBank accession numbers
AGCRV PB01-155	155	America	*vp4, vp6, vp7*	EF589103, EF589105, EF589107
AGCRV	ARV	America	*vp4, vp6, vp7*	NC010589, NC010593, NC010594
GCRV 096	096	Hubei, China	*vp4, vp6, vp7*	JN206664, HQ452490, JN206665
GCRV 104	104	Hubei, China	*vp6*	HM234682
GCRV 097	097	Shanxi, China	*vp4*	GQ469997
GCRV 873	873	Hunan, China	*vp4, vp6, vp7*	AF403392, AF260512, AF260513,
GCRV 875	875	Hubei, China	*vp6, vp7*	AF403412, AF403409
GCRV 876	876	Jiangxi, China	*vp6, vp7*	AF403413, AF403410
GCRV 991	991	Hunan, China	*vp6, vp7*	AF403414, AF403411
GCRV GD108	108	Guangdong, China	*vp4, vp6, vp7*	HQ231208, HQ231205, HQ231203
GCRV HeNan988	988	Henan, China	*vp4, vp6*	KC847325, KC847328
GCRV HN12	H12	China	*vp6*	KC130075
GCRV HS11	H11	China	*vp6*	KC130076
GCRV HuNan794	794	Hunan, China	*vp4, vp6*	KC238681, KC238684
GCRV HZ08	H08	Zhejiang, China	*vp4, vp6, vp7*	GQ896337, GU350746, GU350744
GCRV JS12	J12	China	*vp6*	KC130077
GCRV NC11	N11	China	*vp6*	KC130078
GCRV QC11	Q11	China	*vp6*	KC130079
GCRV QY12	Q12	China	*vp6*	KC130080
GCRV YX11	Y11	China	*vp6*	KC130081
GCRV ZS11	Z11	China	*vp6*	KC130082
GCRV 106	106	China	*vp4, vp6*	KC201171, KC201174
GCRV 918	918	China	*vp4, vp6*	KC201182, KC201185
GCRV JX01	J01	Jiangxi, China	*vp7*	JQ042807
GCRV JX02	J02	Jiangxi, China	*vp7*	JX263303
Bovine rotavirus B223	Bovine		*vp4, vp6, vp7*	D13394, AF317128, X57852

### Virus culture and transmission electron microscopy observation

Cell culture, viral infection and propagation determination were performed as previously described (Fang et al. 1989). GCRV 096 particles were extracted with the differential centrifugation at 250-6000 g, and the supernatant was then ultracentrifuged at 35,000 g at 4°C for 2.5 h. The purified virus pellet was resuspended in phosphate-buffered saline (PBS) with pH 7.4 and then stored at -70°C for the further use. After removing the cell culture medium from the confluent monolayer cell, the monolayer cell was rinsed two times with the PBS buffer and the virus was added with the adsorption for one hour at room temperature. Then, after aspirating off the virus, the maintain solution (M199 containing 2% FBS) was added. The infected CIK cells were incubated at 28°C and observed daily.

Electron microscopic section of the infected CIK cells with CPE was made and observed in transmission electron microscope.

### RT-PCR amplification

With viral RNA kit (Takara, Dalian, China), the GCRV 096 genome RNA was extracted from purified GCRV 096 virus. The cDNA of GCRV 096 genome RNA was acquired with RT-PCR kit (Takara, Dalian, China) using the random primers and M-MLV reverse transcriptase.

According to the genome sequence of GCRV 873, primers for GCRV 096 *vp4*, *vp6* and *vp7* gene amplification were designed based on homologous sequence in GCRV 873: 5″-CACTTCGCACTCTCTCTACAATG-3′ and 5′-AGTACGACACTTCCCGCCGTT-3′, 5′-TGTGATGGCACAGCGTCAG-3′ and 5′-GTTAGA CGAACATCGCCTGC-′3, 5′-TCACCACGATGCCACTTCAC-3′ and 5′-CGGTGCTTAATCGGATGGCT-3′, respectively. Primers were also designed based on homologous sequence from GCRV GD108 and GCRV HZ08 for *vp4*, *vp6* and *vp7* gene: 5′-ACTTACGGCCACTATCATGG-3′ and 5′-TCGGTGTACACGACCTAAG-3′, 5′-CTTTGAGTCGACGCACGTAT-3′ and 5′-CCGTCGGGTGGATTAGGTC-3′, 5′-TCTACTGCCAAGATGGCCAC-3′ and 5′-GCACGCACCTTACTTACAGCA-3′. The PCR cycling conditions were an initial denaturation at 95°C for 3 min followed by 30 cycles consisting of 94°C for 30 s, 55°C for 60 s and 72°C for 70 s, and a final extension step of 30 min at 72°C. The composition of the PCR system (50 μl) includes 33 μl sterile water, 3 μl dNTP (each is 2.5 mmol/L), 10 pmol/L primer for 2 μl each, 10 × buffer for 5 μl (containing Mg++), DNA for 100 ng and Taq polymerase for 0.25 μl (5 U/μl). The aimed genes were purified using Gel Extraction Kit (Takara, Dalian, China) from gelose gel and connected with pMD18-T vector at 16°C, then transformed to DH5α E.*coli*. The recombined plasmid was verified by sequencing.

### Gene sequence analysis of GCRV isolates

Sequences of *vp4*, *vp6* and *vp7* genes were aligned by using the Clustal V method in DNAstar software. Subsequently, the alignment was manually adjusted. Variable sites, information sites, genetic distances, and homologic rates of segments were calculated with MEGA5.1 (Tamura et al. 2007) and DnaSP5.10 (Rozas and Rozas 1999) software.

### Phylogenetic relationships of GCRV isolates

Evolutionary models of *vp4*, *vp6* and *vp7* gene in GCRV were separately simulated in ModelTest3.7 (Posada and Crandall 1998). Subsequently, phylogenetic trees were restructured with simulation results. Using bovine rotavirus B223 as the outgroup, maximum parsimony (MP) trees, maximum likelyhood (ML) trees, and UPGMA trees were constructed with MEGA 4.1 (Tamura et al. 2007) software. MP trees were also built in PAUP4.0 (Swofford 1998) by running the heuristic search with TBR branch swapping, 100 random addition sequence replications, and non-parameter bootstrap resampling procedures to get the coincidence of the resultant MP trees. Bayesian analysis were performed with MrBayes3.12 (Huelsenbeck and Ronquist 2001) using the general-time-reversible + gamma + invariants (GTR + G + I) model of sequence evolution and four Markov Chain Monte Carlo (MCMC) sampling to assess phylogenetic relationships. We set the parameters in MrBayes as follows: nst = 6, rate = gamma, basefreq = estimate, generations = 10,000,000, and the posterior probability and branches of the phylogeny were summed by burnin = 500 and contype = allcompat.

### Sequence variation analysis of *vp4*, *vp6* and *vp7* genes in GCRV isolated to the same genotype

Sequences of *vp4*, *vp6* and *vp7* genes in GCRV isolated to the same genotype were aligned by using the Clustal V method in DNAstar software. Alignment was manually adjusted. Variable sites were analysed.

## Results

### Virus infection in sensitive cellls and particle identification

**Figure 1:**
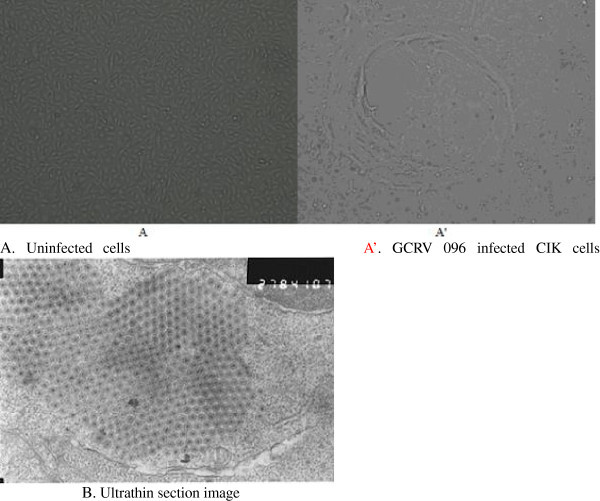
**CPE in the CIK cells 3 d after GCRV 096 isolate inoculation (A, A’ 100×) and Crystalline array of viral particles (B 50,000×).** Notes: **A**. The control CIK cells without GCRV096 inoculation. **A’**. CPE in the CIK cells 3 d after GCRV 096 isolate inoculation. **B**: Crystalline array of viral particles.

Three days after the culture of the CIK cells infected by GCRV 096, CPE phenomenon was observed and the shedding and apoptosis occurred in most of the CIK cells five days after the infection. While, the controlled CIK cells without the infection by the virus grew well (Figure 1).

A large number of virus particles without the envelope structure crystalline in CIK cells were detected from transmission electron microscopy ultrathin section of CIK cells infected with GCRV 096 (Figure 1). The shape, size and the arrangement of GCRV 096 were similar to those of grass carp reovirus (Ke et al. 1990).

### Detection by RT-PCR

*vp4*, *vp6* and *vp7* genes were PCR amplified from GCRV 096, subcloned into a pMD18-T vector and sequenced. The length of *vp4*, *vp6* and *vp7* genes in GCRV 096 was 1981 bp, 1258 bp, and 855 bp, respectively (GenBank accession numbers: JN206664, HQ452490 , and JN206665).

### Sequence analysis

*vp4*, *vp6* and *vp7* genes in the these GCRV isolates contain 184, 447 and 375 informative sites, respectively. Table 2 shows the identity and divergence among GCRV isolates based on *vp4*, *vp6* and *vp7* genes, respectively. Based on the data shown in Table 2, it is apparent that genetic distances of *vp4*, *vp6* and *vp7* genes among GCRV 096, GCRV 873, GCRV 875, GCRV JX01, GCRV 876 and GCRV 991 or AGCRV and AGVRV PB01-155 were small, and their homologous rates were high. Also, genetic distances among GCRV HZ08, GCRV GD108, GCRV 918, GCRV HuNan794, GCRV HeNan988, GCRV 106, GCRV ZS11, GCRV QC11, GCRV HN12, GCRV HS11, GCRV YX11, GCRV JS12, GCRV QY12, GCRV JX02, and GCRV 097 were small, with elevated homologous rates. Other genetic distances were far and the genetic identities were small.

**Table 2:** **Identity (above the diagonal) and divergence (under the diagonal) between GCRV isolates based on the** ***vp4*** **,** ***vp6*** **,** ***vp7*** **gene [×1000]**

	Based on the ***vp4*** gene											Based on the ***vp7*** gene											
GCRV	155	ARV	096	097	873	108	988	794	H08	106	918	GCRV	155	AVR	096	873	875	876	991	108	H08	J01	J02
155		1000	599	317	603	299	293	292	286	293	296	155		1000	203	303	279	287	289	219	214	302	213
ARV	0		599	317	603	299	293	292	286	293	296	ARV	0		203	303	279	287	289	219	214	302	213
096	453	453		295	993	303	299	298	304	298	304	096	1178	1178		212	196	197	197	199	208	213	207
097	946	946	827		311	981	990	987	994	990	987	873	767	767	920		902	999	1000	199	197	994	205
873	445	445	5	827		298	297	296	299	296	302	875	856	856	910	100		901	902	222	208	899	206
108	967	967	886	20	872		970	970	963	971	968	876	776	776	855	1	102		999	204	200	993	197
988	971	971	891	10	882	31		997	986	998	986	991	782	782	848	0	100	1		204	200	995	195
794	977	977	896	13	888	31	3		985	998	985	108	1218	1218	920	885	891	865	870		986	203	987
H08	941	941	875	6	863	35	14	14		986	984	H08	1194	1194	904	863	880	843	848	14		202	998
106	973	973	893	10	884	30	2	2	13		987	J01	772	772	897	6	103	7	5	890	864		193
918	962	962	897	13	888	33	15	15	17	13		J02	1300	1300	913	858	876	839	844	14	2	859	
**Based on the** ***vp6*** **gene**																							
GCRV	155	ARV	096	104	873	875	876	991	108	988	H12	H11	794	H08	J12	N11	Q11	Q12	Y11	Z11	106	918	
155		1000	548	230	548	547	547	548	196	233	241	235	233	204	235	235	235	235	235	233	233	232	
ARV	0		548	230	548	547	547	548	196	233	241	235	233	204	235	235	235	235	235	233	233	232	
096	500	500		220	994	998	998	997	201	242	235	235	241	202	233	233	239	233	233	243	241	241	
104	936	936	769		238	246	246	246	190	232	229	232	232	198	238	238	228	238	232	232	232	223	
873	503	503	6	751		999	999	998	204	243	235	235	242	205	232	232	239	232	232	243	242	242	
875	516	516	3	751	1		1000	999	211	238	231	231	238	203	233	233	236	233	233	238	238	237	
876	516	516	3	751	1	0		999	211	238	231	231	238	203	233	233	236	233	233	238	238	237	
991	514	514	4	755	2	1	1		211	238	231	236	238	200	233	233	236	233	233	238	238	237	
108	1427	1427	1340	1527	1261	1236	1236	1236		207	197	203	207	965	204	204	203	204	197	200	200	199	
988	834	834	682	939	693	665	665	665	1157		975	972	998	208	971	971	990	971	973	998	998	995	
H12	844	844	680	959	692	659	659	659	1214	26		991	975	210	990	990	979	990	992	975	976	973	
H11	850	850	683	962	694	662	662	662	1211	29	9		973	205	998	998	981	998	999	973	974	971	
794	838	838	685	931	697	670	670	670	1149	2	25	28		206	972	972	990	972	974	998	999	996	
H08	1400	1400	1288	1510	1210	1196	1196	1196	32	1113	1165	1162	1106		204	204	200	204	205	206	206	204	
J12	850	850	683	958	694	662	662	662	1228	29	10	2	29	1178		1000	980	1000	998	972	973	970	
N11	850	850	683	958	694	662	662	662	1228	29	10	2	29	1178	0		980	1000	998	972	973	970	
Q11	834	834	689	923	701	670	670	670	1142	10	22	19	10	1099	20	20		980	982	992	991	988	
Q12	850	850	683	958	694	662	662	662	1228	29	10	2	29	1178	0	20	20		988	972	973	970	
Y11	854	854	686	958	698	667	667	667	1219	28	8	1	27	1170	2	2	19	2		974	975	971	
Z11	834	834	682	939	693	665	665	665	1142	2	25	28	2	1099	29	29	8	29	27		999	996	
106	838	838	685	935	697	670	670	670	1149	2	24	27	1	1106	28	28	9	28	26	1		997	
918	851	851	692	946	704	677	677	677	1154	5	28	30	4	1124	31	31	12	31	29	4	3		

### Simulation results of evolutionary model and phylogenetic relationships of GCRV isolates

Simulation results of the GCRV evolutionary model based on *vp4*, *vp6* and *vp7* gene from ModelTest3.7 (Posada and Crandall 1998) are shown in Table 3. The simulation results are used to construct phylogenetic trees.

**Table 3:** **Simulation results of the evolutionary model**

			***vp4*** gene	***vp6*** gene	***vp7*** gene
Model selected:	HKY + G	GTR + G	HKY + G	TVMef + G	K80 + G
-lnL =	10960.6680	10947.8525	10486.8574	10480.6113	6139.4121
K =	5	9	5	8	2
AIC =		21913.7051			20977.2227
Base frequencies:					
freqA =	0.2726	0.2724	0.2561	0.2530	
freqC =	0.2541	0.2476	0.2612	0.2586	
freqG =	0.2361	0.2401	0.2285	0.2313	
freqT =	0.2371	0.2400	0.2541	0.2571	
Substitution model:					
R(a) [A-C] =		1.9847		1.8471	
R(b) [A-G] =		3.8446		7.0961	
R(c) [A-T] =		1.3223		1.3157	
R(d) [C-G] =		1.7384		1.3664	
R(e) [C-T] =		4.4150		7.0961	
R(f) [G-T] =		1.0000		1.0000	
i/tv ratio =	1.3563		2.5439		
Proportion of invariable					
sites =	0	0	0	0	0
Gamma distribution					
shape parameter =	4.2112	4.3849	3.8529	3.7611	3.8868

**Figure 2:**
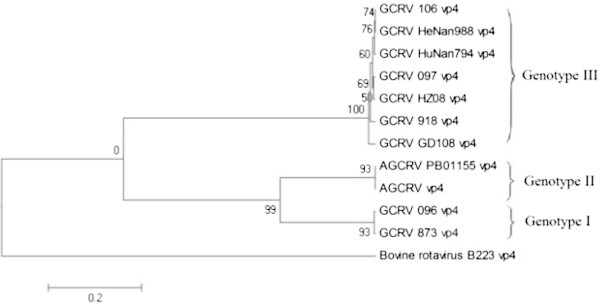
**The constructed UPGMA tree based on the** ***vp4*** **gene (Numbers indicate degree of confidence) was created first in MEGA software and completed with Microsoft Paint program.**

Topological structures of constructed phylogenetic trees, based on *vp4*, *vp6* and *vp7* genes of GCRV in this article are basically coincident. According to evolutionary simulation results, there is the UPGMA tree constructed based on *vp4* gene in Figure 2. The results showed that the cluster on the top of the UPGMA tree consisted of GCRV 106, GCRV HeNan988, GCRV HuNan794, GCRV 097, GCRV 918, GCRV GD108 and GCRV HZ08. The second cluster was AGCRV PB01-155 and AGCRV. The third cluster contained GCRV 096 and GCRV 873.

**Figure 3:**
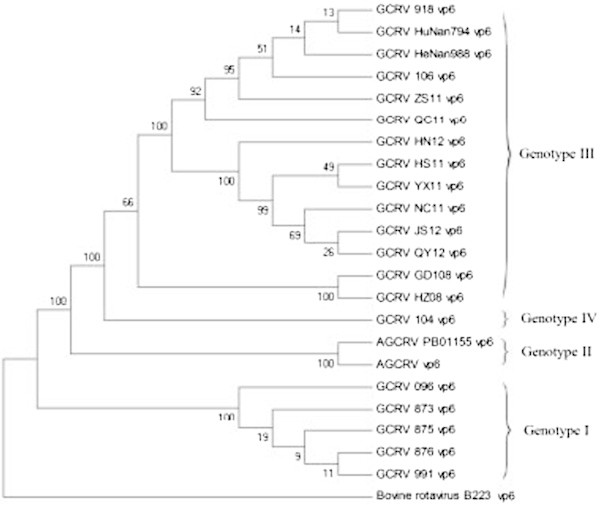
**The constructed MP tree based on the** ***vp6*** **gene (Numbers indicate the degree of confidence) was created first in PAUP software and completed with Microsoft Paint program.**

In Figure 3, the MP tree was constructed based on *vp6* gene. On the MP tree, the cluster on the top consisted of GCRV 106, GCRV HeNan988, GCRV HuNan794, GCRV 918, GCRV ZS11, GCRV QC11, GCRV HN12, GCRV HS11, GCRV YX11, GCRV JS12, GCRV QY12, GCRV GD108 and GCRV HZ08. The second cluster was GCRV 104. The next cluster was AGCRV PB01-155 and AGCRV. The last cluster contained GCRV 096, GCRV 875, GCRV 876, GCRV 991 and GCRV 873.

**Figure 4:**
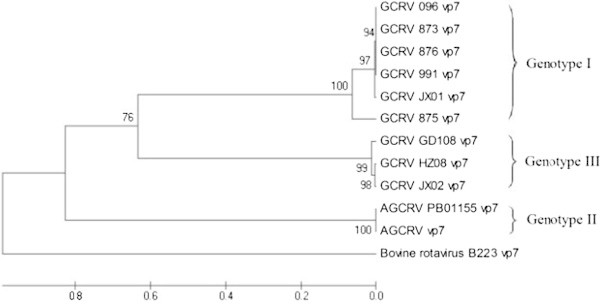
**The constructed UPGMA tree based on the** ***vp7*** **gene (Numbers indicate the degree of confidence) was created first in MEGA software and completed with Microsoft Paint program.**

In Figure 4, the UPGMA tree was constructed based on *vp7* gene. The cluster on the top of this tree consisted of GCRV 096, GCRV 875, GCRV 876, GCRV JX01, GCRV 991 and GCRV 873. The second cluster was GCRV GD108, GCRV HZ08 and GCRV JX02. The last cluster contained AGCRV PB01-155 and AGCRV. The phylogenetic relationships of GCRV096, GCRV 991, GCRV 876, GCRV 873, GCRV JX01, and GCRV 875 or GCRV HZ08, GCRV JX02, and GCRV GD108 are relatively close.

### Sequence analysis of *vp4*, *vp6* and *vp7* genes in GCRV isolated to the same genotype

By analysing variable sites, we found the ratios of variation sites located on the third condon and transitions were respectively 71.6% and 82.1% in *vp4* gene; 57.1% and 80.0% in *vp6* gene; 77.3% and 89.3% in *vp7* gene.

## Discussion

Amongst all aquareovirus isolates, GCRV is one of the most pathogenic agents (Fang et al. 2002). GCRV can cause fatal epidemics of haemorrhagic disease in grass carp, and affects approximately 85% of fingerling and yearling populations (Jiang and Ahne 1989). Many GCRV isolates have been isolated in recent years, and various of them have been reported to exhibit distinctive differences in virulence (Fang et al. 2002). Moreover, new GCRV isolates were found constantly. In this study, GCRV 096 is a new GCRV isolate similar to GCRV 873, GCRV 875, GCRV 876, GCRV 991 and GCRV JX01.

In order to analyse the difference among GCRV isolates as well as their evolutionary relatiohship, it is necessary to genotyping. Currently, uniform criteria in place for virus genotyping are still unavailable. In hepatitis C virus, a more than 30% nucleotide sequence divergence between genotypes is generally considered standard (Simmonds 2004). The genetic heterogeneity among genotypes of hepatitis E virus has been shown to be more than 20% (Schlauder and Mushahwar 2001). In GCRV, relatively conservative *vp4*, *vp6* and *vp7* gene encode major outer capsid proteins and consist of many variable sites (Rangel et al. 1999). So, *vp4*, *vp6* and *vp7* gene could be used for GCRV genotyping.

The genetic distances among GCRV 096, GCRV JX01, GCRV 873, GCRV 875, GCRV 876 and GCRV 991 were small with high homologous rates. Furthermore, these isolates clustered together into one cluster on constructed phylogenetic trees. These results present that GCRV 096, GCRV JX01, GCRV 873, GCRV 875, GCRV 876 and GCRV 991 are attributed to the same genotype, i.e. genotype І. Genetic distances between AGCRV PB01-155 and AGVRV were small and their homologous rates were also high. On phylogenetic trees, AGCRV and AGCRV PB01-155 separately clustered into one cluster. These results indicate that AGCRV and AGCRV PB01-155 are attributed to a new genotype, i.e. genotype II. Genetic distances among GCRV HZ08, GCRV GD108, GCRV 918, GCRV HuNan794, GCRV HeNan988, GCRV 106, GCRV ZS11, GCRV QC11, GCRV HN12, GCRV HS11, GCRV YX11, GCRV JS12, GCRV QY12, GCRV JX02, and GCRV 097 were extremely small with especially high homologous rates. Furthermore, these isolates clustered together into one cluster on phylogenetic trees. GCRV HZ08, GCRV GD108, GCRV 918, GCRV HuNan794, GCRV HeNan988, GCRV 106, GCRV ZS11, GCRV QC11, GCRV HN12, GCRV HS11, GCRV YX11, GCRV JS12, GCRV QY12, GCRV JX02, and GCRV 097 were attributed to another new genotype, i.e. genotype III. In contrast, genetic distances between GCRV 104 and other GCRV isolates were large, and their homologous rates were small. On the phylogenetic tree (Figure 4), GCRV 104 separately clustered into one cluster. GCRV 104 is attributed to a new genotype, i.e. genotype IV.

The genotyping results obtained are consistent with previous research conclusions. The study of Wang indicated there were different genotypes of GCRV in China (Wang et al. 2012a). The biological characteristics of GCRV isolates belonging to the same genotype indicated they were analogous. For example, in an artificial infection test, GCRV HZ08 and GCRV GD108 can cause mortality of 60–80% of the yearly grass carp (approx. 10 cm in length), without obvious CPE in CIK cells (Ye et al. 2012; Zhang et al. 2010b). However, American grass carp reovirus (AGCRV) is not strongly connected with infectious disease in fish, although it is commonly detected by cell culture during routine inspections of healthy fish (Goodwin et al. 2010). GCRV 873, GCRV 096, GCRV 875, GCRV 876, GCRV 991 and GCRV JX01 can arouse significant CPE in CIK cells (Zhang et al. 2010a; Wang et al. 2012b). Furthermore, other characteristics of these two isolates were also similar. The genomic segments pattern of GCRV 875 was found to be similar to that of GCRV 873 (Fang et al. 2002). Polyacrylamide gel electrophoresis atlases of GCRV 873, GCRV 875, GCRV 876 and GCRV 991 were also the same (Fang et al. 2002).

The comparative analysis of the geographic location (Table 1) of collected GCRV isolates together with the difference between GCRV isolates and GCRV genotyping indicated there was no obvious relationship between the evolution of GCRV and geographical distribution of GCRV. In the same genotype, the ratios of variation sites on the third condon and the transitions in *vp4*, *vp6* and *vp7* gene were high.

Hemorragic disease of grass carp outbreaks seriously in China. Many isolates of grass carp reovirus have been discovered while new isolates are being isolated constantly. The systematic difference comparison of the different GCRV isolates has not been reported. In this study, we have verified the diference among various GCRV genotypes. GCRV genotyping has important significance to diagnosis and treatment in hemorrhagic disease of grass carp, especially to vaccine development. Comparison of different GCRV isolates and genotyping are helpful to further our understanding in GCRV genetic variation and evolution and the development of more effective preventative strategies against GCRV.

This study provides a foundation for revealing differences among GCRV isolates. Simultaneously, it is significant for the further research on genetically engineered vaccines against grass carp haemorrhagic disease and grass carp breeding for disease resistance.

## Abbreviations

bp: Base pairs
CIK: Grass carp kidney cell line
CPE: Cytopathic effect
dNTPs: Deoxynucleotide triphosphates
FBS: Fetal bovine serum
GCRV: Grass carp reovirus
MgCl2: Magnesim chloride
min: Minute
ML: Maximum likelihood
MP: Maximum parsimony
PBS: Phosphate-buffered saline
PCR: Polymerase chain reaction
RT-PCR: Reverse transcription-PCR
TBR: Tree-bisection-recorrnection.
